# E2F8 is a nonreceptor activator of heterotrimeric G proteins

**DOI:** 10.1186/1750-2187-2-3

**Published:** 2007-03-30

**Authors:** Ian S Hagemann, Kirk D Narzinski, Thomas J Baranski

**Affiliations:** 1 Departments of Medicine and of Molecular Biology & Pharmacology, 660 S. Euclid Ave., Campus Box 8127, St. Louis, Missouri 63110, USA

## Abstract

**Background:**

Heterotrimeric G proteins are important for numerous signaling events in eukaryotes, serving primarily to transduce signals that are initiated by G protein-coupled receptors. It has recently become clear that nonreceptor activators can regulate the level of heterotrimeric G protein signaling and, in some cases, drive cycles of receptor-independent G protein activation. In this study, we used a yeast expression cloning strategy to identify novel nonreceptor activators of heterotrimeric G proteins in a human adipocyte cDNA library.

**Results:**

The human transcription factor E2F8 was found to activate heterotrimeric G proteins, suggesting a specific biological role for this recently described member of the E2F family. Epistasis studies showed that E2F8 acted at the level of G proteins and was specific for G*α*_i _over Gpa1. E2F8 augmented receptor-driven signaling, but also activated G proteins in the absence of a receptor. The GTPase-activating protein RGS4 antagonized the effect of E2F8, showing that E2F8's effect on G*α *involved nucleotide turnover. The entire E2F8 protein was required for full activity, but the majority of the signaling activity appeared to reside in the first 200 residues.

**Conclusion:**

In yeast, E2F8 is a guanine nucleotide exchange factor (GEF) for the *α *subunit of heterotrimeric G proteins. The molecular mechanism and biological significance of this effect remain to be determined.

## Background

Cells use heterotrimeric G proteins to transduce a wide variety of signals, including polypeptide hormones, small molecules, odorants, and light. They are thus one of the primary means by which cells gather information about their environment [1]. In the conventional paradigm for G protein signaling, a ligand-activated G protein-coupled receptor (GPCR) is responsible for catalyzing the exchange of GTP for GDP on the heterotrimer's *α *subunit. This exchange results in the dissociation of the heterotrimer, releasing the *α *and *βγ *moieties to exert cell- and context-dependent downstream effects. It has generally been thought that activated receptors are the primary source of guanine nucleotide exchange factor (GEF) activity for heterotrimeric G proteins. However, in recent years it has become apparent that several nonreceptor GEFs also exist, providing cells with a further level of control over G protein activity.

One of the earliest nonreceptor GEFs to be identified was GAP-43, also known as B-50 or neuromodulin. GAP-43 is abundant in neuron growth cones and promotes axonal pathfinding during development and regeneration [2]. Investigating the function of GAP-43, Fishman and coworkers found that it had GEF activity toward G*α*_o_, another protein that is abundant in growth cones, and that this activity was synergistic with that of receptors [3-8].

More recently, a series of papers has described a diverse collection of "activators of G protein signaling" (AGS proteins). AGS1 has intrinsic GEF activity [9,10], and the mechanism of this activity appears to be analogous to the GEF activity of receptors, since receptors and AGS1 can compete for heterotrimers [11]. AGS2–8 activate G protein signaling in yeast, but appear to do so by disrupting heterotrimers rather than by stimulating nucleotide exchange on G*α *[12].

The biological role of nonreceptor GEFs has been only minimally elucidated, but a recent series of experiments has defined a compelling function for one such molecule in a specific developmental context. Ric-8A/synembryn is a nonreceptor GEF with specificity for G_o_, G_i_, and G_q _[13]. *C. elegans *zygotes undergo an asymmetric first division that requires Ric-8 for correct positioning of the mitotic apparatus [14-16]. Ric-8 appears to be the GEF that drives a receptor-independent cycle of GTP binding and hydrolysis, wherein each iteration of the cycle incrementally repositions the mitotic spindle [17].

In the course of a screen for novel nonreceptor activators of G proteins, we have found that human E2F8 promotes nucleotide exchange on G*α *subunits. This finding was unexpected, since E2Fs have previously been considered to be primarily transcription factors, modulators of apoptosis, and recruiters of chromatin remodeling complexes. Here, we present evidence that the functions of E2F8 include augmentation of heterotrimeric G protein signaling, thus potentially linking two signaling pathways that have heretofore been considered to be separate.

## Results

### E2F8 selectively activates G*α*_i _in yeast

#### Identification of E2F8 by expression cloning

**Figure 1:**
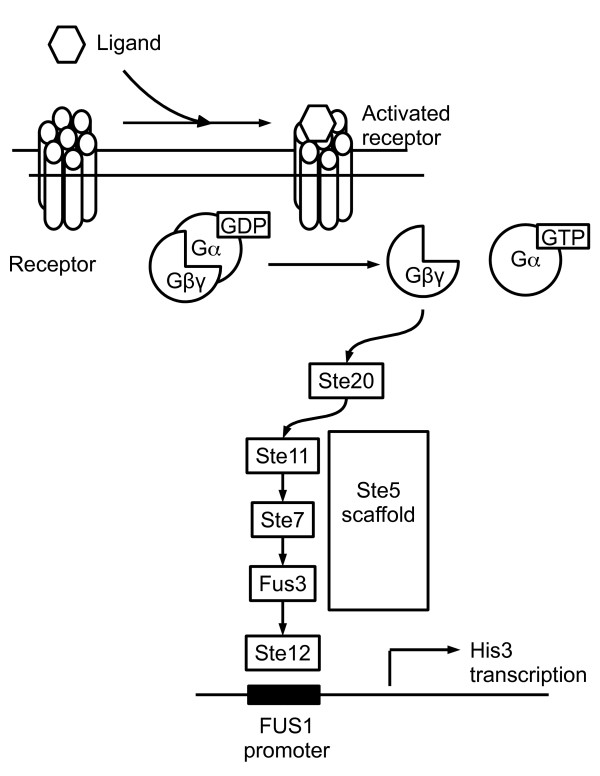
**Yeast G protein/MAP kinase reporter cascade**. In *S. cerevisiae *strains BY1142 and BY1173, activation of an episomally expressed mammalian GPCR stimulates nucleotide exchange on a chimeric G*α *subunit. The newly free *βγ *moiety activates a MAP kinase cascade that leads to transcription of the HIS3 reporter gene, allowing growth on histidine-deficient medium.

We screened a human adipocyte cDNA library to identify novel activators of heterotrimeric G proteins. The screen made use of an engineered *S. cerevisiae *strain (BY1142) in which activation of the G protein/MAP kinase mating pathway [18] resulted in transcription of the HIS3 gene, allowing yeast to grow on medium lacking histidine (Figure 1) [19]. Plasmids conferring histidine prototrophy were isolated from yeast colonies and sequenced; the phenotype was then confirmed by retransforming each plasmid into yeast and replica-plating on selective His^- ^plates containing 3-amino-1,2,4-triazole (3-AT). Since 3-AT is a competitive inhibitor of the His3 gene product, the maximal tolerated concentration of 3-AT reflects the strength of activation of the reporter.

The screen identified several cDNAs that would have been expected to cause G protein or MAP kinase activation, including three splicing variants of the lysophosphatidic acid receptor [20] and a constitutively active MEKK2 [21], thus validating our methodology. We also isolated three identical clones encoding full-length E2F8 [22-24].

To confirm the signaling function of E2F8, we replica-plated yeast expressing E2F8, E2F8 fused to the yellow fluorescent protein (YFP), or empty vector onto selective plates containing 0–100 mM 3-AT. E2F8- and E2F8-YFP-expressing yeast were able to grow on up to 100 mM 3-AT, the highest concentration tested (Table 1), whereas the empty vector control permitted only minimal growth of yeast on 1 mM 3-AT. A constitutively active mutant of the complement factor 5a receptor (C5aR NQ) was used as a positive control; this receptor allowed growth on up to 10 mM 3-AT, as reported previously [25].

**Table 1:** G protein activation by E2F8

ADE2 plasmid	Signaling	Fluorescence
E2F8	+++++++	-
E2F8-YFP	+++++++	+
C5aR NQ	++++	-
Vector	-	-

Yeast BY1142, which expresses a chimeric Gαi3,Gpa11−41
 MathType@MTEF@5@5@+=feaafiart1ev1aaatCvAUfKttLearuWrP9MDH5MBPbIqV92AaeXatLxBI9gBaebbnrfifHhDYfgasaacH8akY=wiFfYdH8Gipec8Eeeu0xXdbba9frFj0=OqFfea0dXdd9vqai=hGuQ8kuc9pgc9s8qqaq=dirpe0xb9q8qiLsFr0=vr0=vr0dc8meaabaqaciaacaGaaeqabaqabeGadaaakeaacqqGhbWriiGacqWFXoqydaWgaaWcbaGaeeyAaKMaeG4mamJaeiilaWIaee4raCKaeeiCaaNaeeyyaeMaeeymaeZaaSbaaWqaaiabigdaXiabgkHiTiabisda0iabigdaXaqabaaaleqaaaaa@3B69@ subunit, was transformed with the indicated ADE2 plasmids along with empty URA3 vector. Transformants were replica-plated onto Ura^- ^His^- ^Ade^1 mM ^plates containing varying concentrations of 3-AT. Yeast were assessed for growth after 3 days. The maximal concentration of 3-AT tolerated by the transformants is indicated: 100 mM (+++++++), 50 mM (++++++), 20 mM (+++++), 10 mM (++++), 5 mM (+++), 2 mM (++), 1 mM (+), or 0 mM (-). Fluorescence was determined by examining yeast colonies on a dissecting microscope and is reported as either + (fluorescent colony) or – (non-fluorescent colony). C5aR NQ, a constitutively active mutant of the C5a receptor, was used as a positive control.

**Figure 2:**
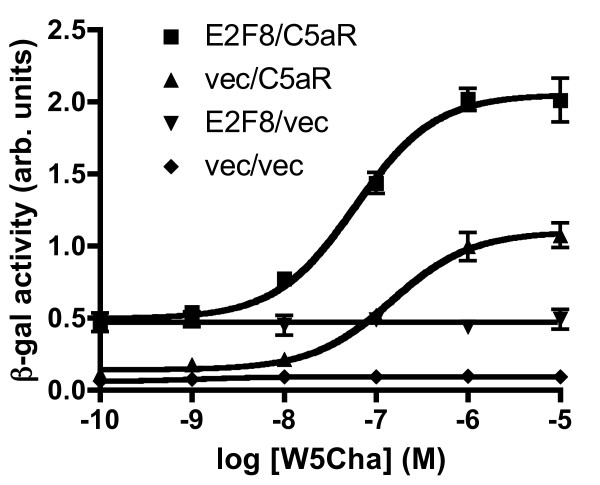
**E2F8 effect on receptor-mediated signaling**. Yeast BY1142 was transformed with a P_FUS1_-LacZ reporter construct (TRP1 selection), then transformed with plasmids encoding E2F8 or empty vector (ADE2 selection) and C5aR or empty vector (URA3 selection). The agonist W5Cha was used to activate the receptor. After four hours of ligand treatment, cells were lysed and *β*-galactosidase activity was measured by colorimetry. This experiment is representative of three independent trials, each of which examined three transformants per condition.

E2F8 activation of G protein signaling was verified by using an episomal *β*-galactosidase reporter assay in place of the yeast growth assay. We have previously described reconstitution of signaling by the human complement factor 5a receptor (C5aR) in yeast [25]. The receptor can be activated by a small-molecule agonist, W5Cha, that is able to cross the yeast cell wall. When E2F8 was expressed in this system, the reporter was constitutively activated, and this effect did not compete with the receptor-dependent signal (Figure 2). E2F8 had a small but statistically significant effect on the EC_50 _for receptor activation by W5Cha, which was 150 nM in the absence of E2F8 (95% CI, 120–170 nM) and 59 nM in the presence of E2F8 (95% CI, 52–67 nM). These results show that E2F8 is a receptor-independent means of activating G proteins, but is also able to augment receptor-mediated signaling.

#### Epistasis mapping of E2F8 function

The signaling activity of E2F8 could in theory arise from activation at any level of the G protein/MAP kinase reporter cascade (Figure 1), including transcriptional upregulation of cascade components. To map E2F8's site of action, we tested it for signaling in yeast strains that were deficient for specific components of the cascade (Table 2). We found that E2F8 was downstream from receptors, since it signaled in yeast BY1142, which lacks the endogenous Ste3 gene (a-mating factor receptor). On the other hand, E2F8 was upstream of Ste4^G*β*^, Ste11^MAPKKK^, and Ste12 (MAP kinase substrate). In yeast lacking any of these components, E2F8 failed to activate transcription of the HIS3 reporter. It therefore appeared that E2F8 activated the G protein/MAP kinase cascade at a point no lower than G proteins. These screens were performed in the absence of any receptor, so E2F8 must either act directly on G proteins or act to increase the cellular level of free G*βγ*.

**Table 2:** Epistasis mapping and G protein specificity of E2F8 signaling

Yeast strain	G*α *subunit	Reporter cascade mutations	ADE2 plasmid	Signaling
BY1142	G*α*_i3,Gpa1(1–41)_	Intact reporter cascade	E2F8	+++++++
			Vector	-
BY1187	G*α*_i3,Gpa1(1–41)_	ΔSte4^G*β*^	E2F8	-
			Vector	-
BY1206	G*α*_i3,Gpa1(1–41)_	ΔSte11^MAPKKK^	E2F8	-
			Vector	-
BY1207	G*α*_i3,Gpa1(1–41)_	ΔSte12 (MAPK substrate)	E2F8	-
			Vector	-
BY1173	Gpa1_DCGLF_	Intact reporter cascade	C5aR NQ	++++
			E2F8	-
			Vector	-

A plasmid encoding E2F8 or empty vector (ADE2 selection) and empty URA3 vector were transformed into the indicated yeast strains. Transformants were replicated onto selective plates containing 3-AT and assessed for growth after 3 days. The strength of signaling is reported as in Table 1.

Alternatively, the activity of E2F8 may require a small amount of upstream basal activity, which it can then amplify at a downstream point. The G protein specificity of E2F8 (described below) argues against this possibility.

#### G protein specificity of E2F8

To further characterize the site of action of E2F8, we reasoned that if E2F8 interacted directly with G*α*, it might show selectivity for particular G*α *isoforms. To test this hypothesis in the yeast system, we measured signaling by E2F8 in yeast strains expressing different G*α *subunits.

All of the yeast strains used in our studies contained yeast/human chimeric G*α *subunits. Wild-type mammalian G proteins do not signal when expressed in yeast because they cannot couple to downstream components of the yeast G protein/MAP kinase cascade. Chimeric G*α *subunits have the advantage that they can be activated by mammalian receptors, but can also interact with yeast G*βγ*[25,26].

Strain BY1142 contains a G*α *subunit consisting of 41 residues of the endogenous yeast G*α *(Gpa1) followed by residues 32–354 of human G*α*_i3 _(yielding the chimera G*α*_i3,Gpa1(1–41)_). In this strain, E2F8 strongly activated the MAP kinase cascade (Table 2).

BY1173 contains instead an *α *subunit (Gpa1_DCGLF_) identical to Gpa1 except for its last five residues, which are derived from human G*α*_i_. The chimerism of Gpa1_DCGLF _allows it to interact with G*α*_i_-coupled receptors, but in the absence of a receptor this G protein is functionally identical to Gpa1. E2F8 showed no activity when expressed in BY1173 (Table 2), indicating that it was selective for G*α*_i3 _over Gpa1. These findings do not necessarily define the endogenous G protein coupling of E2F8, but rather indicate that E2F8 is specific for particular G*α *isoforms and must therefore act at the level of G*α*.

#### RGS4 inhibition of E2F8 signaling

We wished to determine whether E2F8 catalyzed nucleotide exchange on G*α *or, instead, served to disrupt G*αβγ *heterotrimers, for example by binding to G*α*-GDP and sequestering the G*α *subunit away from G*βγ*. Either of these mechanisms would increase free G*βγ*, which is responsible for signaling in the yeast mating cascade. To differentiate between these two possibilities, we determined whether E2F8's effect was antagonized by coexpression of RGS4. The GTPase-activating protein RGS4 catalyzes nucleotide hydrolysis on G*α*_i_-GTP [27], thus inactivating the protein and promoting re-association of heterotrimers. If E2F8 served merely to displace G*βγ*, then its effect would be indifferent to the nucleotide binding state (GTP or GDP) of G*α*.

In yeast BY1142, E2F8 alone allowed yeast to grow on up to 100 mM 3-AT, but when RGS4 was co-expressed, the signaling was reduced to 1 mM (Table 3). Thus, G protein activation by E2F8 requires nucleotide cycling on G*α*. As a control, we verified that the signaling activity of a constitutively active GPCR (C5aR NQ [28]) was antagonized by fluorescently tagged RGS4, consistent with the receptor's ability to catalyze nucleotide binding on G*α*_i_. The simplest interpretation of these data is that E2F8 increases free G*βγ *by promoting GTP-for-GDP exchange on G*α*. It seems unlikely that E2F8 merely stabilizes the GTP-bound state, as it can activate G proteins in the absence of receptors or any other guanine nucleotide exchange factor (GEF).

**Table 3:** Effect of RGS4 on E2F8 signaling

ADE2 plasmid	URA3 plasmid	Signaling	Fluorescence
C5aR NQ	Vector	++++	-
	RGS4-GFP	+	+
E2F8	Vector	+++++++	-
	RGS4-GFP	+	+
Vector	Vector	-	-
	RGS4-GFP	-	+

Yeast BY1142 was transformed with the indicated plasmids. Three transformants per condition were replicated onto selective plates containing 3-AT and assessed for growth after 3 days. The strength of signaling and fluorescence is reported as in Table 1.

### Structure/function analysis of E2F8

**Figure 3:**
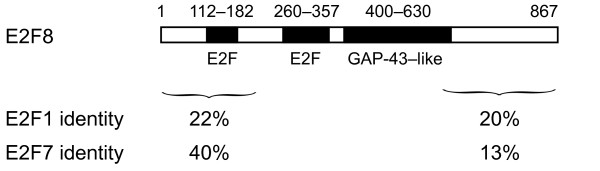
**Domain structure of E2F8**. E2F8 includes two E2F homology domains, both of which contain the RR*X*YD consensus motif that interacts directly with DNA. The region 400–630 has 16% amino acid identity with GAP-43, a known activator of heterotrimeric G proteins. Approximate boundaries of the homology regions are indicated. The percentage of amino acid identity to human E2F1 and E2F7 is indicated for the regions 1–200 and 610–867.

E2F8's ability to activate G proteins was highly surprising, as this would be a novel function for a member of the E2F family. We examined the sequence of E2F8 to identify any motifs that might shed light on the behavior we observed. The primary structure of E2F8 contained two E2F DNA-binding domain at positions 112–182 and 260–357; each of these regions included the canonical RR*X*YD motif that interacts directly with DNA [29] (Figure 3). The remaining regions of E2F8 exhibited only minimal similarity to other known proteins, as determined by BLAST searching [30].

In light of E2F8's surprising ability to activate G proteins, we asked whether it was similar to other known proteins with this function. E2F8 lacks the G protein regulatory (GPR) sequence that appears to define the G*α*-GDP-stabilizing AGS proteins [31]. Intriguingly, we found that E2F8 did contain an extended region of homology to GAP-43, a known nonreceptor GEF: between residues 400 and 630, E2F8 was 27% similar to GAP-43 and 16% identical. On the other hand, the homology domain did not include the first ten amino acids of GAP-43. These residues of GAP-43 are sufficient to activate G_o_, albeit with low potency [7].

**Figure 4:**
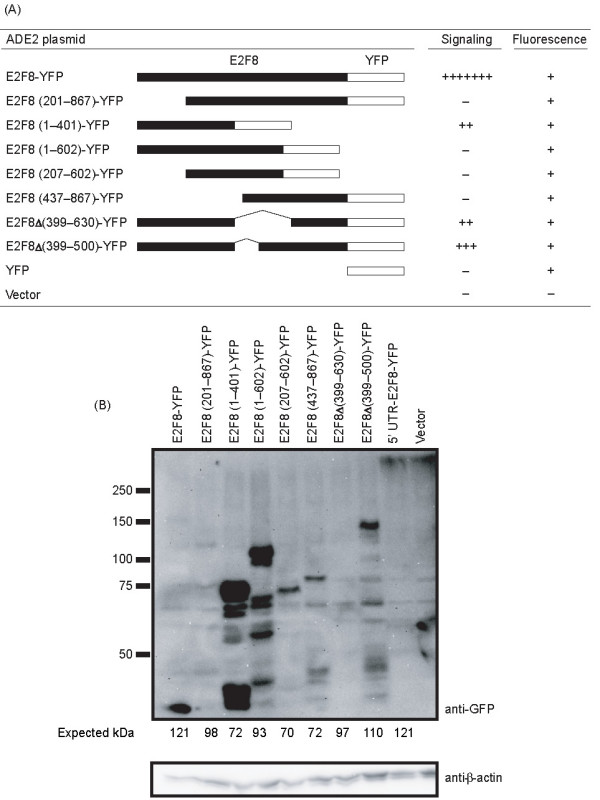
**Structure/function analysis of E2F8**. (A) Yeast BY1142 was transformed with the indicated E2F8 mutants (ADE2 selection) along with empty URA3 vector. Transformants were replicated onto selective plates containing 3-AT and assessed for growth after 3 days. The strength of signaling and fluorescence is reported as in Table 1. (B) Yeast cells expressing E2F8 mutants were grown in liquid culture, lysed, and examined by immunoblotting using an anti-GFP (top) antibody. The membrane was stripped and reprobed using an anti-*β*-actin antibody (bottom) as a loading control. The 5' UTR-E2F8-YFP construct contained the wild-type 5' UTR in addition to E2F8; the other full-length construct lacked the UTR.

We constructed a series of E2F8 truncation and deletion mutants to identify regions of the protein that were required for function. The mutants were fused to YFP to allow expression to be verified. Truncation of the first 200 amino acids of E2F8 eliminated its ability to activate G proteins (Figure 4A), suggesting that the N-terminal domain was required for the activity. Consistent with this hypothesis, E2F8 residues 1–401 were sufficient to activate the reporter pathway, although they did so less strongly than the full-length protein. Interestingly, the E2F8 (1–602)-YFP construct was not able to activate G proteins. The truncation may in this case have resulted in misfolding, or residues 402–602 may have autoinhibitory functions. Two E2F8 constructs in which the GAP-43 homology domain was completely (E2F8 Δ(399–630)-YFP) or partially (E2F8 Δ(399–500)-YFP) deleted did retain signaling ability, suggesting that the ability to activate G proteins did not reside in this domain. Since none of the mutants had full activity, it appeared that the entire protein was required for wild-type signaling in this system, but that the majority of the activity resided in the N-terminal region.

The variable signaling by E2F8 mutants did not simply reflect failure of some mutants to be expressed in yeast. All of the YFP-tagged mutants resulted in yeast fluorescence, arguing that the proteins were expressed (Figure 4A). Most of the mutants could be detected by immunoblotting, but their apparent levels were quite variable, reflecting either variable expression, degradation of the proteins during yeast lysis, or insufficient sensitivity of our technique (Figure 4B). There was, however, no correlation between strength of signaling and detection of the protein by immunoblot. E2F8-YFP, which signaled strongly, was only faintly detected, whereas E2F8 (1–602)-YFP, which did not signal, was strongly detected.

Importantly, all of the constructs that failed to signal were detected at least weakly by immunoblot, showing that they were expressed-particularly since these contructs also rendered yeast fluorescent. We cannot, however, exclude the possibility that different expression levels contributed somewhat to the different phenotypes we observed with the mutants.

## Discussion

We identified the transcription factor E2F8 in the course of a screen for novel activators of heterotrimeric G proteins. In *S. cerevisiae*, E2F8 was able to activate a G protein/MAP kinase reporter pathway; this activity was specific for particular G protein isoforms, mapped epistatically to the level of heterotrimers, and was antagonized by a GTPase-accelerating protein. The amino-terminus of the protein appeared to be most important for G protein activation. The most parsimonious interpretation of these results is that E2F8 stimulates nucleotide exchange on the *α *subunit of heterotrimeric G proteins. Since E2F8 did not reduce the maximal receptor-mediated signal (Figure 2), but rather caused a left-shift in the receptor's dose-response curve, E2F8 does not compete with receptors for G proteins. Instead, E2F8 and receptors may be complementary to one another in their mode of action, using different molecular mechanisms to activate G*α*.

G protein activation would be a novel function for an E2F transcription factor. The E2F family of transcription factors has classically been associated with cell-cycle regulation and cell differentiation [32,33]. The "activating" subgroup, E2F1–3, consists of proteins that are bound by pRb; the E2F moiety is released to serve as a transcription factor when pRb is phosphorylated by cyclin/Cdk complexes. E2F4 and 5 also bind to pocket proteins (pRb, p107, and p130) but act primarily to repress E2F target genes by occupying promoters and remodeling chromatin, allowing cells to maintain G0 arrest.  E2F6 lacks the transactivation-related and pocket protein-binding domains of E2F1–5, but appears to have functions similar to the other repressive E2Fs [32,34]. E2F1–6 all bind to DNA most efficiently as an E2F/DP heterodimer [35].

E2F8's apparent ability to activate G proteins may account for its having arisen over the course of evolution. Although both E2F7 and E2F8 have been shown to repress a subset of E2F-regulated genes and reduce cell proliferation, no special function has yet been shown for them. It may be significant that the newer E2Fs are structurally dissimilar to the previously described and well-characterized E2F1–6; unlike these, both E2F8 and E2F7 lack a DP dimerization domain and can bind to DNA efficiently in the absence of DP [22-24,36-38]. In this respect they are similar to the *Arabidopsis *proteins E2L1–3 [39].

Furthermore, they lack a pocket protein interaction domain and therefore are unlikely to interact with pRb, p107, or p130. In yeast, we found that murine E2F7 did not activate G proteins, but we were unable to verify that the protein was expressed (data not shown).

It is curious, but not inconceivable, that a transcription factor should also activate heterotrimeric G proteins. Cell-surface receptors are the primary means by which cells acquire information about their hormonal environment, and these hormonal cues frequently trigger signaling cascades that culminate in a transcriptional response. Many of these cascades use heterotrimeric G proteins as a signaling intermediate, and integrating G protein regulation with transcriptional activity may be important for the coordination of complex cellular processes. For example, the Tubby protein, whose loss causes obesity and retinal degeneration in mice [40], is both a transcriptional regulator and an effector of G_q_ signaling.

As one possible model of E2F8 function, it could be the case that E2F8 serves to set a basal level of G protein activity that is then modulated by receptor activity. The level of E2F8 activity on G proteins could itself be controlled by redistributing E2F8 between the nucleus and cytosol. Since E2F8 has three nuclear localization sequences (NLSes) [22] and has transcriptional effects, it presumably is localized to the nucleus at some times, but it must also at times be located in other compartments if it is to have effects on heterotrimeric G proteins. It is also possible, in a second model, that E2F8 participates in a receptor-dependent G protein activation event, although receptors were not necessary for E2F8 activity in the yeast system. E2F8 could facilitate receptor-stimulated G protein activation, then undergo relocalization to the nucleus to exert transcriptional effects. Thus E2F8 would be both the agent and the object of a signaling event, and a cell-surface receptor could have a relatively direct influence on the transcription of E2F-responsive genes. We do not yet have sufficient data to conclusively test these models.

It is not known whether the observed activity of E2F8 is physiologically significant in mammalian cells, as opposed to the heterologous yeast system. The yeast growth assay has been described numerous times in the literature, including in previous studies of nonreceptor G protein activators such as the AGS proteins [9]. However, we cannot exclude the possibility that the E2F8/G protein interaction may reflect some unexpected feature of the yeast system. The yeast G protein cascade differs from the canonical mammalian cascade in that free G*βγ *is the active signaling moiety. The epistasis and G protein specificity data presented here would, however, seem to rule out a direct transcriptional role of E2F8 in yeast (such as E2F8 upregulation of G*β*). Furthermore, a human E2F protein (E2F1) was previously found to be unable to bind to *S. cerevisiae *genomic DNA [41], again arguing against transcriptional effects.

Numerous future experiments are needed to more fully characterize the role of E2F8 in G protein activation and cellular signaling. The present data are essentially genetic in nature and would be strengthened by complementary biochemical evidence. *In vitro *studies with purified E2F8 and G protein subunits would allow the hypothesis of a direct effect to be tested and would allow G protein specificity to be more thoroughly determined. G protein-E2F8 binding studies would also show more conclusively whether a physical interaction can occur. Further effort should be devoted to determining whether E2F7 or the plant E2Fs also interact with G proteins, as this activity may not be limited to E2F8.

Finally, the influence of E2F8 on G proteins was identified in yeast, but this interaction must next be studied in a more physiologically relevant system. One obstacle to studies in mammalian tissue culture is that E2F8 appears to cause cell-cycle arrest when overexpressed [22-24]. Likewise, in our efforts to make stably transfected mammalian cell lines, E2F8 was lost within a few passages (data not shown). An inducible expression system could be a useful workaround, but it would be equally valuable to investigate the role of E2F8 in a system where it natively plays a demonstrable biological role. Given that E2F8 was cloned from a mature human adipocyte cDNA library, adipose tissue appears to be an interesting starting point for this effort.

## Conclusion

In yeast, E2F8 served as a strong activator of nucleotide exchange on the G*α *subunit of heterotrimeric G proteins. The effect was inhibited by coexpression of RGS4, appeared to occur directly at the level of G proteins, and was primarily derived from residues 1–200 of E2F8. Future studies are needed to determine whether this effect is important *in vivo *and to delineate its molecular mechanism.

## Methods

### Yeast strains

Yeast strain BY1142 has genotype *MATα far1*Δ*1442 tbt1-1 *P_*FUS*1_*-HIS3 ste14::trp1::LYS2 ste3*Δ*1156 *G*α*_i3,Gpa1(1–41) _*his3 leu2 lys2 trp1 ura3 can1 ade2*. Yeast BY1173 has genotype *MATa his3 leu2 trp1 ura3 can1 gpa1::ade2::3 × HA far1::ura3 fus1::*P_*FUS*1_*-HIS3 LEU2::*P_*FUS*1_*-lacZ sst2::ura3 ste2::G418*^R ^*trp1::*GPA1_DCGLF_. Yeast strains with deletions of *STE4 *(BY1187), *STE11 *(BY1206), or *STE12 *(BY1207) were derived from BY1142 by gene replacement in our laboratory. Yeast were transformed by the lithium acetate procedure or by electroporation and were grown on selective media at 30°C. Fluorescence of yeast colonies was determined by examining them at 500× magnification on an Olympus SZX12 dissecting microscope with a mercury-arc light source (Hitschfel Instruments) and YFP or GFP filter set (Olympus).

### Plasmids and cloning

The human adipocyte library (gift of Robin Weinberg, Pharmacia Corp..) consisted of mature human adipocyte cDNA cloned into vector pBN975 under the ADH promoter with ADE2 selection. The library consisted of approximately 3 × 10^6 ^clones. When 24 unselected clones were analyzed, 22 contained an inserted cDNA, with average insert size 1200 bp; the inserts ranged in size from 500 bp to 3000 bp. The plasmid encoding RGS4 fused to the green fluorescent protein (GFP) was a gift of Maurine Linder (Department of Cell Biology and Physiology, Washington University). The constitutively active NQ mutant of the C5a receptor has been described previously [25].

The E2F8-YFP plasmid was constructed by homologous recombination in yeast. The polymerase chain reaction (PCR) was used to construct a DNA fragment containing the sequence of YFP with a 5' arm of homology to E2F8 and a 3' arm of homology to the library vector. These inserts were transformed into yeast BY1142 along with a double-cut parent vector in a 1:1 molar ratio. Constructs were isolated from yeast by plasmid rescue and verified by sequencing (Protein and Nucleic Acid Chemistry Laboratory, Washington University School of Medicine).

E2F8 mutants were also constructed by homologous recombination in yeast. In all cases, an E2F8 insert with 5' ADH promoter homology and 3' YFP homology was recombined into a double-cut YFP vector. For the truncations, PCR was used to synthesize inserts containing the desired E2F8. Recombination was performed as described above. The deletion mutants were constructed by a two-step PCR strategy. In the first step, PCR products encoding E2F8 (1–398) and E2F8 (630–867), or E2F8 (1–398) and E2F8 (501–867), were amplified with arms of homology to the vector and to each other. In the second step, these products were used as templates for an overlap extension PCR reaction. The full-length PCR products were then used for homologous recombination as described above.

### Library screening

The human adipocyte library was screened by the yeast growth assay (described below) to identify cDNAs that activated the G protein/MAP kinase mating cascade. A total of 1.7 × 10^6 ^transformants were screened. cDNAs conferring growth were isolated by plasmid rescue and retransformed into yeast to reconfirm the phenotype. Confirmed positives were then sequenced.

### G protein signaling assays in yeast

For the growth assay, selected yeast colonies were streaked onto a master plate (Ura^- ^Ade^-^), then replica-plated onto selective plates (Ura^- ^His^- ^Ade^1 mM^) containing increasing concentrations of 3-AT (Sigma). The minimal concentration of adenine in the selective plates allowed us to use color selection to ensure that signaling was plasmid-dependent: if a reporter pathway mutation arose — allowing plasmid-independent growth — and the ADE2 plasmid was lost, yeast still grew on minimal adenine medium, but developed a red color that allowed those colonies to be excluded from consideration [42].

For *β*-galactosidase assays, yeast were grown in liquid culture and seeded into microtiter plates (Costar) at an OD_600 _of 0.015 units/cm. The small molecule W5Cha [43] was used as an agonist. After a four-hour incubation at 30°C in the presence of ligand, a lysis/substrate buffer was added (final composition 0.5% Triton X-100, 1 mg/ml chlorophenol red *β*-d-galactopyranoside, 25 mM PIPES, pH 6.8) and color development was allowed to proceed for fifteen hours, then was stopped by addition of Na_2_CO_3 _to 0.2 M. Color was assessed by measuring OD_570 _on a Bio-Rad plate reader, model 680.

### Immunoblotting

For yeast studies, yeast were grown overnight in selective media to an OD_600 _of 1.5 units/cm. Cells were harvested from 1 ml of culture and lysed by vortexing at room temperature for 5 min with 100 *μ*l of acid-washed beads (0.5 mm diameter) and 150 *μ*l of 1× SDS sample buffer containing PMSF (100 *μ*g/ml) and aprotinin, leupeptin, and pepstatin (all 1 *μ*g/ml). A 1 min centrifugation step (1000 × g) was used to clarify the lysate, and the supernatant was used for immunoblotting.

For immunoblots, proteins were separated on SDS-PAGE and transferred to PVDF using a wet transfer apparatus (Bio-Rad). Membranes were blocked with 5% milk in phosphate-buffered saline with 0.1% Tween-20 for 1 hour, then probed with primary and secondary antibodies. Rabbit anti-GFP antibody (Santa Cruz) was used at 1:200 dilution and mouse anti-*β*-actin (ab8224, Abcam) at 1:800. Secondary antibodies were goat anti-rabbit or goat anti-mouse horseradish peroxidase conjugates (Santa Cruz) at 1:10,000 dilution. A chemiluminescent substrate (Lumi-Light Plus, Roche) was applied for five minutes, and signal was recorded using a FluorChem 8900 detection system (Alpha Innotech Corp.).

## Declaration of competing interests

The authors declare that they have no competing interests.

## Authors' contributions

TJB provided project leadership and financial support. Experiments were designed by all three authors and performed by KDN and ISH. ISH wrote the manuscript, which all authors read and approved.
